# Layer-by-layer assembly of procyanidin and collagen promotes mesenchymal stem cell proliferation and osteogenic differentiation *in vitro* and *in vivo*

**DOI:** 10.1093/rb/rbac107

**Published:** 2022-12-26

**Authors:** Zhibiao Bai, Kai Hu, Zeyu Shou, Jiahuan Yu, Hongming Meng, Han Zhou, Liangyan Chen, Tiantian Yu, Ruofei Lu, Na Li, Chun Chen

**Affiliations:** Department of Orthopaedics, The First Affiliated Hospital of Wenzhou Medical University, Wenzhou 325000, P.R. China; Wenzhou Medical University, Wenzhou 325000, P.R. China; Wenzhou Medical University, Wenzhou 325000, P.R. China; Wenzhou Medical University, Wenzhou 325000, P.R. China; Wenzhou Medical University, Wenzhou 325000, P.R. China; Wenzhou Medical University, Wenzhou 325000, P.R. China; Wenzhou Medical University, Wenzhou 325000, P.R. China; Wenzhou Medical University, Wenzhou 325000, P.R. China; Wenzhou Key Laboratory of Perioperative Medicine, Oujiang Laboratory (Zhejiang Lab for Regenerative Medicine, Vision and Brain Health), Wenzhou Institute, University of Chinese Academy of Sciences, Wenzhou 325001, P.R. China; Xinjiang Technical Institute of Physics and Chemistry, Chinese Academy of Sciences, Urumqi 830011, P.R. China; Xinjiang Technical Institute of Physics and Chemistry, Chinese Academy of Sciences, Urumqi 830011, P.R. China; Wenzhou Key Laboratory of Perioperative Medicine, Oujiang Laboratory (Zhejiang Lab for Regenerative Medicine, Vision and Brain Health), Wenzhou Institute, University of Chinese Academy of Sciences, Wenzhou 325001, P.R. China; Department of Orthopaedics, The First Affiliated Hospital of Wenzhou Medical University, Wenzhou 325000, P.R. China; Wenzhou Medical University, Wenzhou 325000, P.R. China

**Keywords:** bone marrow mesenchymal stem cells, osteogenesis, collagen, procyanidine, coating

## Abstract

Collagen, commonly used in tissue engineering, is widespread in various tissues. During bone tissue regeneration, collagen can stimulate the cellular response and determine the fate of cells. In this work, we integrated collagen type II with procyanidin (PC) onto an implant coating by applying a layer-by-layer technique to demonstrate that collagen and PC can participate in the construction of new biomaterials and serve as multifunctional components. The effects of PC/collagen multilayers on the viability of cocultured bone marrow mesenchymal stem cells (BMSCs) were analyzed by cell counting kit-8 analysis and phalloidin staining. The reactive oxygen species level of BMSCs was revealed through immunofluorescent staining and flow cytometry. Osteogenesis-related genes were detected, and *in vivo* experiment was performed to reveal the effect of newly designed material on the osteogenic differentiation of BMSCs. Our data demonstrated that in BMSCs PC/collagen multilayers accelerated the proliferation and osteogenic differentiation through Wnt/β-catenin signaling pathway and enhanced bone generation around the implant in the bone defect model of rabbit femurs. In summary, combination of collagen and PC provided a new sight for the research and development of implant materials or coatings in the future.

## Graphical abstract

**Figure:**
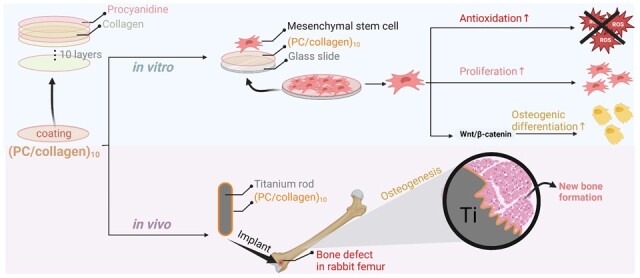

## Introduction

A huge global market for orthopedic implants is well known to exist. In the past few decades, despite great progress in implant materials and surgical methods, the implant failure rate is still at a high level of ∼10–20%, which places enormous pain and economic burdens on many patients [1]. The failure of orthopedic implants is due mainly to the poor bone regeneration ability of the implant surface and its poor osteointegration with the surrounding tissue [2]. After implantation, the interfacial interactions between orthopedic implants and the surrounding biological environment can directly affect biomedical performance [3]. Properly designed bioactive coatings favoring osteogenesis can have a massive impact on osteointegration, which provides a great opportunity to improve the outcomes of orthopedic implants [4].

Extracellular matrix (ECM) protein has been extensively explored as a bioactive coating on orthopedic implants due to its activity in supporting cell survival, adhesion, migration and proliferation [5]. Collagen, as the structural protein of the ECM, is widespread in various tissues, such as cartilage, bone, tendon, skin, cornea and blood vessels, and is available on a large scale [6]. Importantly, some specific amino acid sequences in collagen, such as arginine–glycine–aspartic acid (RGD) [7], can stimulate the cellular response and determine the fate of cells along with bone tissue regeneration. Fixing collagen via physical adsorption or chemical covalence onto implants has been demonstrated to enhance bone generation in different animal models, such as the mandible of dogs, pelvis of sheep, femur and tibia of rabbits [8].

Maintaining bone homeostasis is crucial to bone regeneration, providing the dynamic balance between three key bone cells, osteoblasts, osteocytes and osteoclasts, through a variety of signaling pathways, such as bone morphogenetic protein and macrophage colony-stimulating factor [9, 10]. Reactive oxygen species (ROS), as intracellular secondary messengers, execute many normal functions, including apoptosis and the activation of cell signaling cascades. In normal cells, ROS, as byproducts of energy-producing reactions, occur primarily in mitochondria. The intracellular level of ROS was deliberately equilibrated by the pair of electron transport (reduced nicotine adenine dinucleotide/ozidized nicotine adenine dinucleotide (NADH)/NAD^+^) and the couples of ROS scavenging (reduced nicotine adenine dinucleotide phosphate/oxidized NADPH (NADPH/NADP^+^), reduced glutathione/oxidized glutathione (GSH/GSSG)) [11]. Around the implants, there is an overstressed ROS environment caused by the release of debris from long-term implants, clots in blood during surgery, etc. [12]. The elevated ROS microenvironment has been demonstrated to favor osteoclast differentiation but not osteoblast maturation [13–15]. Presently, well-engineered antioxidant surfaces have been reported to be effective in enhancing bone healing [13].

To better control the properties of the implant surface, many coating technologies have been developed, including spraying and etching. Of these technologies, the layer-by-layer (LBL) technique has showed great potential in maintaining the biological characteristics of the incorporated component, with simple preparation process and independence of the shape of the implant [16]. Most importantly, the abundant coatable materials and well-controlled deposited thickness at the nanoscale by LBL are particularly popular in engineering implant surfaces [17].

Polyphenols, characterized by multiple phenol units on one aromatic ring in its molecular structure, are a large family of compounds naturally obtained from ample plants [18]. More than 8000 polyphenols with diverse structures have been identified [19]. Procyanidine (PC), a specific class of flavonoids, can be extracted on a large scale and exhibits excellent antioxidant activity [20]. In this work, we integrated collagen type II with PC onto an implant coating by the LBL technique to address the above issues. Our data demonstrated that in bone marrow mesenchymal stem cells (BMSCs), PC/collagen coating was beneficial to cell proliferation and osteogenic differentiation and could enhance new bone generation around the implant in a rabbit femur model.

## Experimental section

### Materials and methods

Proanthocyanidin (PC) was purchased from Macklin. Collagen type II from mice tail, Dulbecco’s modified Eagle’s medium (DMEM), Phosphate-buffered saline (PBS) and trypsin were bought from Sigma. Fetal bovine serum (FBS), rhodamine phalloidin (phalloidin-TRITC) and 4′,6-diamidino-2-phenylindole (DAPI) were purchased from Beyotime. Cover slips and silicon wafers were cleaned by piranha solution containing 70% H_2_SO_4_ and 30% H_2_O_2_, for about 4 h at above 90°C, thoroughly dried by air flow.

### Preparation of (PC/collagen)_*n*_ coating

The PC/collagen coating onto various substrates (silicon wafers, titanium rod and glass coverslips) was fabricated by the typical LBL protocol, as reported [21]. Briefly, the substrate was alternatively dipped into PC solution (1 mg/ml) and collagen solution (1 mg/ml) until the desired coating number (*n*), and noted as (PC/collagen)_*n*_. The substrate was thoroughly washed by water and dried by air steam generally. The pH value of PC solution was the same as collagen solution, and buffered with 100 mM Bis–Tris for pH 6.0 or Tris for pH 7.5 and 9.5. HCl (0.2 M) and NaOH (0.2 M) were used to adjust the pH value.

Ellipsometry analysis was used to detect the thickness of newly designed coating in dry state, and quartz crystal microbalance with dissipation monitoring (QCM-D) was used to monitor the growth of the coating during fabrication. These devices were used according to the user’s instructions [13]. By LBL method mentioned previously, the (PC/collagen)_*n*_ coating was deposited with injection velocity 50 μl/min.

The energy dissipation (Δ*D*) and the resonance frequency (Δ*F*) were detected and recorded under different overtones and the data when *ν* = 3 was presented. Ellipsometry was carried out after the film was dried at 50°C for at least 8 h.

Stability test: building block by incubating (PC/collagen)_8_ into predesigned DMEM, PBS or DMEM containing trypsin (0.5 mg/ml). Through ellipsometry analysis the change of thickness after incubation time of 0, 3, 7, 14 and 21 days was detected.

### Cell culture and treatment

The sterile glass slide with diameter 14 mm coated with (PC/collagen) film was placed in a 24-well plate, irradiated with ultraviolet light for 2 h, and then ∼5000 cells/well BMSCs were implanted in each well. The cells were cultured in complete medium containing 10% FBS and in a humidified incubator with 5% CO_2_, at 37°C. The medium was replaced every 2–3 days.

### Cell proliferation assays

The viability of BMSCs cultured on coated slides with different thickness was detected by Cell Counting Kit 8 (CCK-8) analysis (Dojindo Molecular Technologies, Japan). Briefly, 5000 BMSCs were planted in each well of a 24-well plate. At Days 1, 3, 7 and 14 of cell growth, 10 µl of CCK-8 reagent was added at 100 µl complete medium and incubated in a constant temperature foster box at 37°C for 2 h. Finally, the absorbance at 450 nm wavelength was detected by a microplate reader (Molecular Devices, SPECTRA Max Plus, USA). Each group was repeated three times.

### Immunofluorescence detection of TRITC phalloidin

After treatment, the glass slides in 24-well plates were taken out and fixed with 4% paraformaldehyde (Biosharp, China) for 10 min at room temperature. Cells were placed in 0.1% Triton X-100 (Beyotime Biotechnology, China) to increase cell membrane permeability for 15 min, then sealed with 1% BSA (Beyotime Biotechnology, China) for 30 min. Then, cells were labeled with 200 μl per slide TRITC-phalloidin for 30 min. The nuclei of BMSCs were stained with DAPI of 5 μg/ml. After each step, the cells were washed with PBS for three times. Finally, the slides were placed on the slides with drops of anti-fluorescence quench agent and taken picture under the fluorescence microscope (Leica, Germany) immediately. The corresponding excitation/emission filter was selected according to the manufacturer’s instructions [22].

### ROS detection

BMSCs were cultured in 24-well plates containing glass slides with or without coated with (PC/collagen) coating. When the cell density reached about 80%, H_2_O_2_ (0 or 200 μM) was used to stimulate BMSCs for 6 h, then 2′,7′-dichlorodihydrofluorescein diacetate (DCFH-DA) (10 μM), the ROS detector bought from Beyotime Biotechnology was added at 37°C for 20 min, then the cells were washed with PBS and collected. Fluorescence was detected by flow cytometry (CytoFLEX, USA) and data were analyzed using FlowJo V10 software (Tree Star Inc, USA). To measure ROS by fluorescence microscopy, the cell culture procedure was the same as before, DCFH-DA staining for 20 min at 37°C. The glass slides were removed from the plate and analyzed through a fluorescence microscope.

### RNA isolation and quantitative real-time polymerase chain reaction

According to the manufacturer’s instructions, the total RNA of the BMSCs was extracted using RNA Isolation Kit with Spin Column (Beyotime Biotechnology, China) [23]. cDNA was reverse-transcribed in a 20-µl reaction system using the qRT Master Mix (Toroivd, China). Using SYBR Green qPCR Master Mix (Toroivd, China), the cDNA was used to perform real-time polymerase chain reaction (qRT-PCR) analysis on a QuantStudio5 real-time PCR system (ThermoFisher, USA). The absorbance of each well was measured after 40 cycles of 95°C for 10 s and 60°C for 30 s. The expression of related mRNA was analyzed with the formula 2^−ΔΔCt^. All primers used in this study were ordered from Sangon Biotech (Shanghai, China).

### Animal test

All animal operations and protocols were approved by the Experimental Animal Center of Wenzhou Medical University (SYXK 2015-009). Each five New Zealand rabbits aged 5–6 months with almost the same area of bone defect in femoral condyle were treated with uncoated and coated titanium rods, respectively. The surgical procedure for implanting titanium rods was carried out under general anesthesia by intravenous injection of 20% ethyl carbamate. Approximately 2 cm skin incision on the lateral side of the hind leg was made to expose the lateral condyle of femur. After the muscle and periosteum were separated, the bone defect (2.15 mm in diameter and 10 mm in depth) was created by drilling perpendicular to the femur. Subsequently, a cylindrical titanium rod (with or without coating) was implanted into the area of bone defect. The incision was closed with surgical sutures after povidone-iodine disinfection. After 4 and 8 weeks, the rabbits were sacrificed by intravenous injection of air to collect the femur samples. The samples were immersed in 4% paraformaldehyde and then tested by micro-CT with a scanning voltage source of 80 kV, a current of 300 μA, and a 360° rotation (rotate 0.6°/step) [13]. Two hundred micrometers from the titanium implant surface was selected and reconstructed to reveal the newly formed bone around these rods. From the reconstructed images, the total bone volume (TV) and new bone volume (BV) were calculated.

### Statistical analysis

All numerical data were expressed as mean ± standard deviation (SD) and were analyzed by SPSS 22 (IBM, USA). Unpaired Student’s t-test was used to analyze the differences between two groups and ANOVA followed by Dunnett’s test was used to for the comparisons of multiple groups. The histograms were made with Origin2022b (OriginLab, USA). A *P*-values <0.05 was considered significant. The *, ** and *** in figures indicated *P*-values <0.05, 0.01 and 0.001, respectively.

## Results and discussion

### Preparation of PC/collagen multilayers

**Figure 1.**
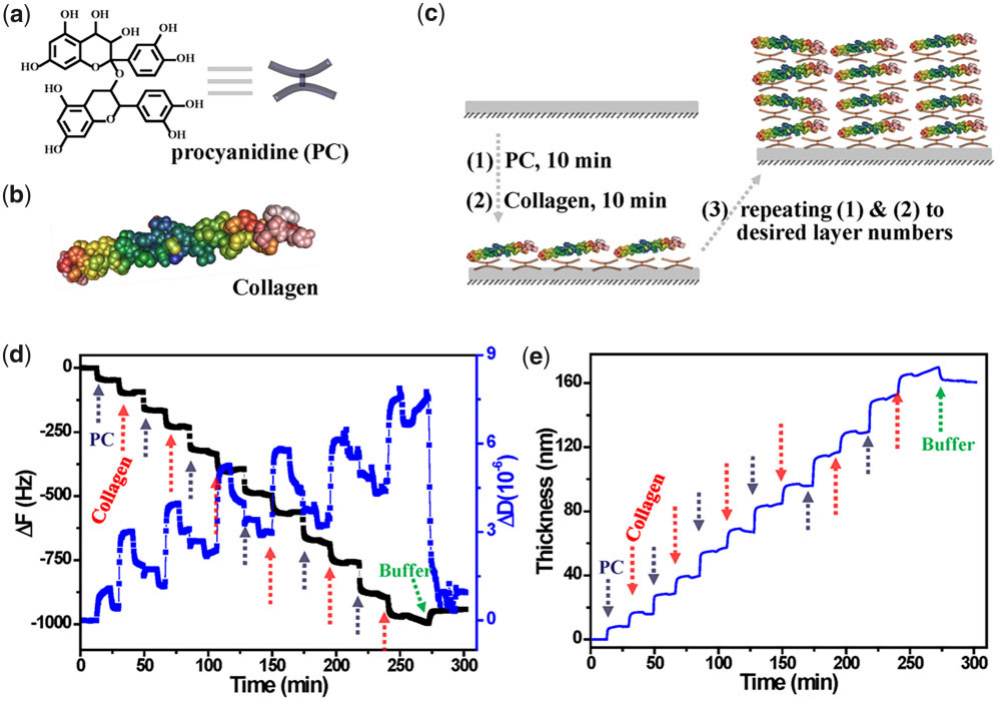
(**a**) Schematic illustration of the fabrication of PC/collagen by LBL process. (**b**) The shifts of Δ*F* and Δ*D* monitored by QCM-D during fabricating (PC/collagen)_6_ at pH 8.5. (**c**) The thickness of PC/collagen multilayers deposited onto QCM-D sensor at pH 8.5.

As schematically illustrated in Fig. 1a, the PC/collagen coating was fabricated by alternatively dipping the substrate in the solutions of PC and collagen with the concentration of 1 mg/ml in the environment with pH of 8.5. QCM-D was utilized to monitor the process of coating. As shown in Fig. 1b, the decreasing Δ*F* and increasing Δ*D* observed during the process of coating indicated the continuous deposition of PC and collagen. The thickness of deposited PC/collagen was obtained by the Voigt model in the affiliated software of QCM-D and displayed a step-by-step increase with the addition of PC and collagen (Fig. 1c). The plateau at the end of the LBL process was the washing by PBS buffer, suggesting a good stability of deposited PC/collagen during washing. It was revealed by QCM-D test that the thickness of (PC/collagen)_6_ was about 162 nm.

**Figure 2.**
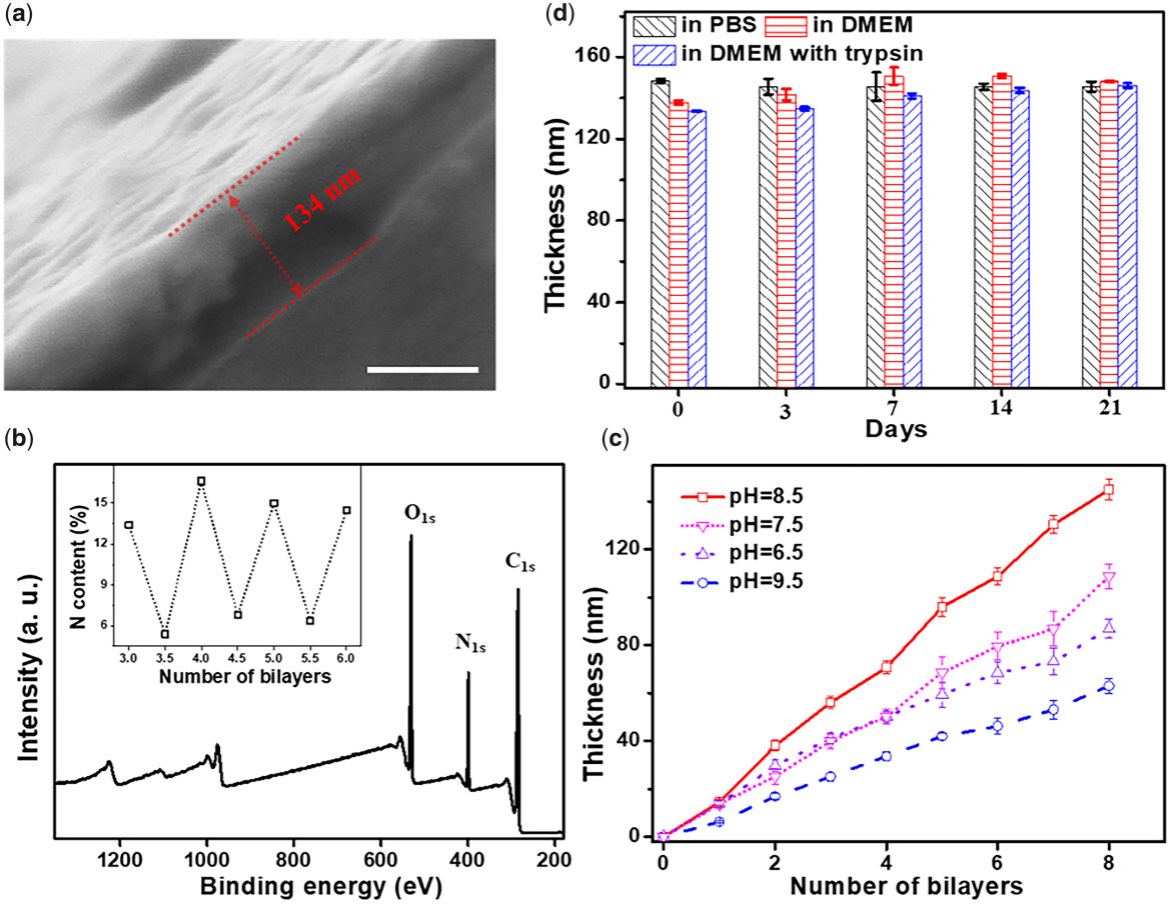
(**a**) SEM images of the cross-section of (PC/collagen)_8_ fabricated at pH 8.5. (**b**) XPS analysis of (PC/collagen)_4_. The content (%) of N was dependent on the outermost material (the integers in the *x*-axis are the outermost layers of the PC, the other points were the outermost layers of collagen) of PC/collagen fabricated at pH 8.5. (**c**) The relationship between the thickness of PC/collagen coating and the number of bilayers prepared at different pH values. (**d**) The thickness of (PC/collagen)_8_ fabricated at pH 8.5 after incubating in different solutions for different days. The scale bar is 100 nm.

When the cross-section of the coating was scanned by scanning electron microscope (SEM), it was observed that PC/collagen was deposited sequentially and uniformly distributed on the surface of silicon substrate (Fig. 2a). Under SEM, the thicknesses of (PC/collagen)_8_ were ∼134 nm (Fig. 2a). Another direct evidence is the N signal in the XPS spectrum of the (PC/collagen)_8_ coating (Fig. 2b). There is no Si signal at all, suggesting that the (PC/collagen)_8_ coating completely covered the substrate with a thickness that exceeded the detection limit of XPS. In addition, the curve of the N content (%) in the coating displayed oscillation dependent on the outermost material (a relatively low N content for the PC outermost layer and a high N content for the collagen outermost layer) during the LBL process (inset of Fig. 2b), which further illustrated that PC/collagen has been deposited successfully with the LBL method. The PH value of the environment had a great influence on the fabrication of the coating (Fig. 2c), and the optimal pH was 8.5. Stability was key to cellular behavior and was evaluated before any cellular experiment by immersing (PC/collagen)_8_ into DMEM, PBS and DMEM containing trypsin for three weeks at 37°C. The thickness of (PC/collagen)_8_ recorded on preset days showed no obvious difference with incubation time, indicating its superstability (Fig. 2d). The thickness tested by ellipsometry was much lower than the thickness tested by QCM-D, e.g. for (PC/collagen)_6_ fabricated at pH 8.5, the thickness was only 109 nm by ellipsometry, while it was ∼162 nm by QCM-D. Such a difference might be due to the thickness tested by ellipsometry being in the dry state vs the hydrated state for QCM-D.

**Figure 3.**
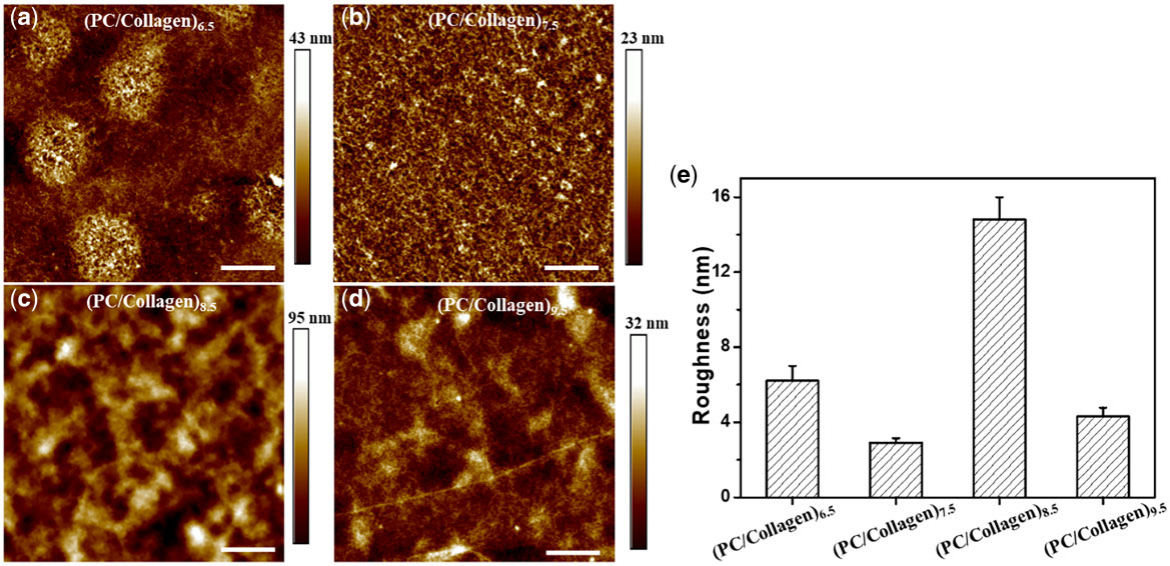
AFM Images of (PC/collagen)_8_ fabricated at different pH values (**a**) 6.5, (**b**) 7.5, (**c**) 8.5 and (**d**) 9.5. (**e**) The roughness of these coatings.

Surface morphology has a significant effect on the cytological behavior of BMSCs, like proliferation and differentiation [24]. The morphologies characteristics of (PC/collagen)_8_ prepared at different pH values were observed by atomic force microscopy (AFM). As exhibited in Fig. 3a–d, the coatings were able to attach evenly beneath the substrates. However, no regular surface topography information was observed. In the roughness analysis, the roughness was maximum at pH 8.5, ∼15.8 nm. Based on a previous report, increased roughness was beneficial to BMSC attachment, proliferation, and differentiation when the roughness was lower than 98.3 nm [25]. Combining the above results, pH 8.5 was selected for PC/collagen fabrication, at which point PC/collagen had the fastest growth and highest roughness.

### PC/collagen coating promoted the proliferation and antioxidant activity of BMSCs

**Figure 4.**
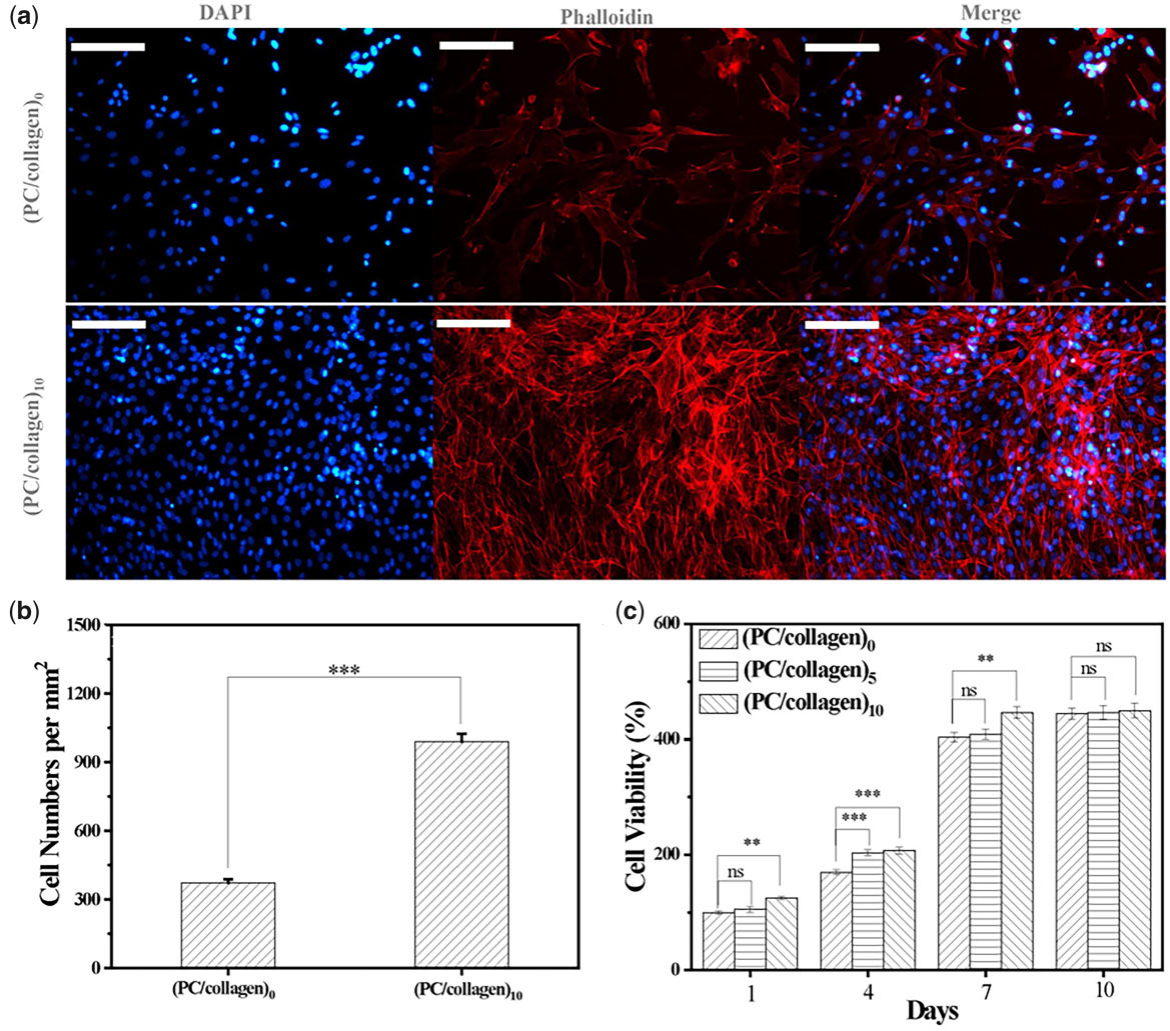
(**a**) Immunofluorescence staining of BMSCs cocultured with or without (PC/collagen)_10_ for 7 days with DAPI and phalloidin (**b**) the comparison of the stained BMSCs in (a). (**c**) the viability of BMSCs cocultured onto (PC/collagen)_n_ (*n* = 0, 5, 10) was detected by CCK-8 method. The scale bars in (a) are 200 μm. (*n* = 3; ns, no significance; *P*-values <0.01 and 0.001 were marked with ** and ***, respectively).

Since oxidative stress is closely related to cell growth, an ideal material was expected to reduce the level of intracellular ROS and promote the proliferation. In this study, the effects of newly designed materials on the proliferation of cocultured BMSCs were detected by phalloidin staining and CCK-8 analysis, with ROS levels analyzed by flow cytometry and immunofluorescent staining. On the seventh day, immunofluorescence staining for nuclei (blue) and f-actin (red) of BMSCs of the (PC/collagen)_10_ and (PC/collagen)_0_ groups were performed (Fig. 4a). The cell numbers were counted and compared between the two groups, consistent with the CCK-8 test (Fig. 4b). Cell proliferation analysis showed that the BMSCs cultured in slides coated with (PC/collagen)_10_ for 7 days exhibited higher cell viability than those of (PC/collagen)_5_ group and control group (PC/collagen)_0_, slides without coating (Fig. 4c). It has been previously reported that proanthocyanidins can promote the proliferation of co-cultured cells when the concentration reaches 100 μg/ml, which is consistent with the results of this study [26]. This positive effect was probably related to the protective effect on the cell membrane and the inhibitory effect on CASP-1-induced pyroptosis of the planted cells [27]. Collagen was able to promote the proliferation of cocultured cells, such as chondrocytes, which may be related to its ability to promote communication between cultured cells and cytoplasm macromolecules, thereby regulating cell adhesion and proliferation [28].

**Figure 5.**
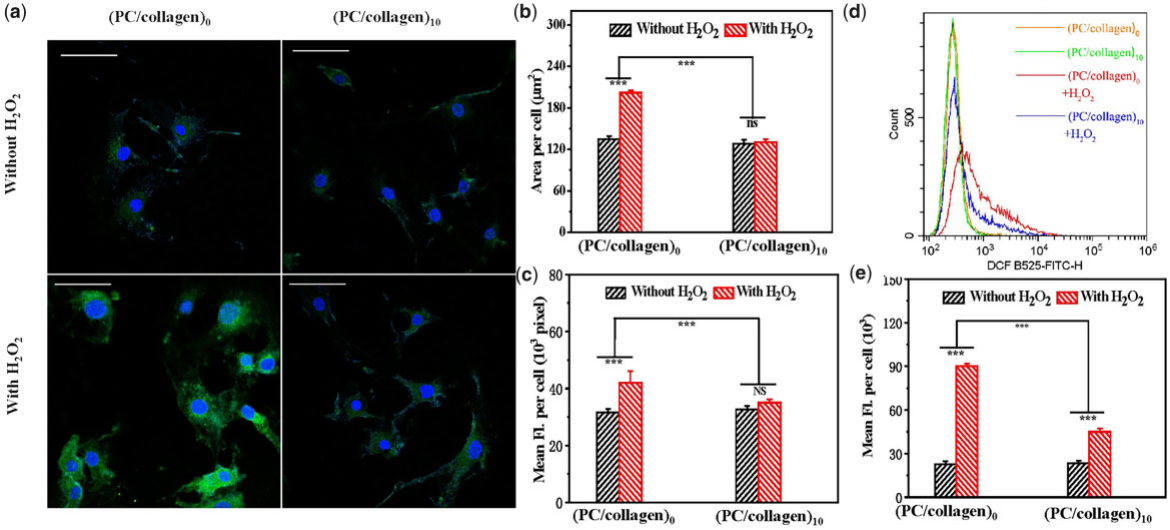
(**a**) The intracellular ROS level of BMSCs cultured in coated or uncoated slides with or without treatment of H_2_O_2_ was detected by fluorescent staining. comparison of (**b**) the average positively stained area and (**c**) mean fluorescence intensity (fl.) of single cell. (**d**) The average fluorescence intensity of cells in (a) detected by flow cytometry. (**e**) Comparison of data from (d). (*n* = 3; ns, significance; *P*-value <0.001 was marked with ***). The scale bars in (a) are 50 μm.

To explore the antioxidant properties of this newly designed material, hydrogen peroxide was used to interfere with cells cultured on different materials to promote intracellular ROS production. As shown in Fig. 5, the concentration of hydrogen peroxide used in this study did not cause cell death in a large area. Under the intervention of hydrogen peroxide, the green fluorescence intensity of the intracellular ROS probe cocultured without materials was significantly stronger than the green fluorescence intensity of BMSCs cocultured with materials and untreated with hydrogen peroxide (Fig. 5a–c). We also quantitatively compared the average fluorescence intensity of each cell, and the results were consistent with the results observed under the fluorescence microscope (Fig. 5c). The quantitative results of ROS detection by flow cytometry also revealed that the newly designed materials had obvious antioxidant effects (Fig. 5d and e). ROS are the product of cell metabolism, which in turn affects cell metabolic activity, intracellular signal transduction, cell function and fate [29]. When excessive ROS accumulate, they can destroy the cell membrane, proteins, DNA and other cellular components, leading to cell senescence and apoptosis [30]. Some functional foods containing proanthocyanidins, such as grape seed proanthocyanidins extract (GSPE), also have antioxidant activity [31]. In addition, proanthocyanidins have also been reported to play an antiosteoporotic role by stimulating bone formation through their antioxidant effect [32].

### The PC/collagen multilayers promoted osteogenic differentiation of BMSCs by activating the Wnt/β-catenin

**Figure 6.**
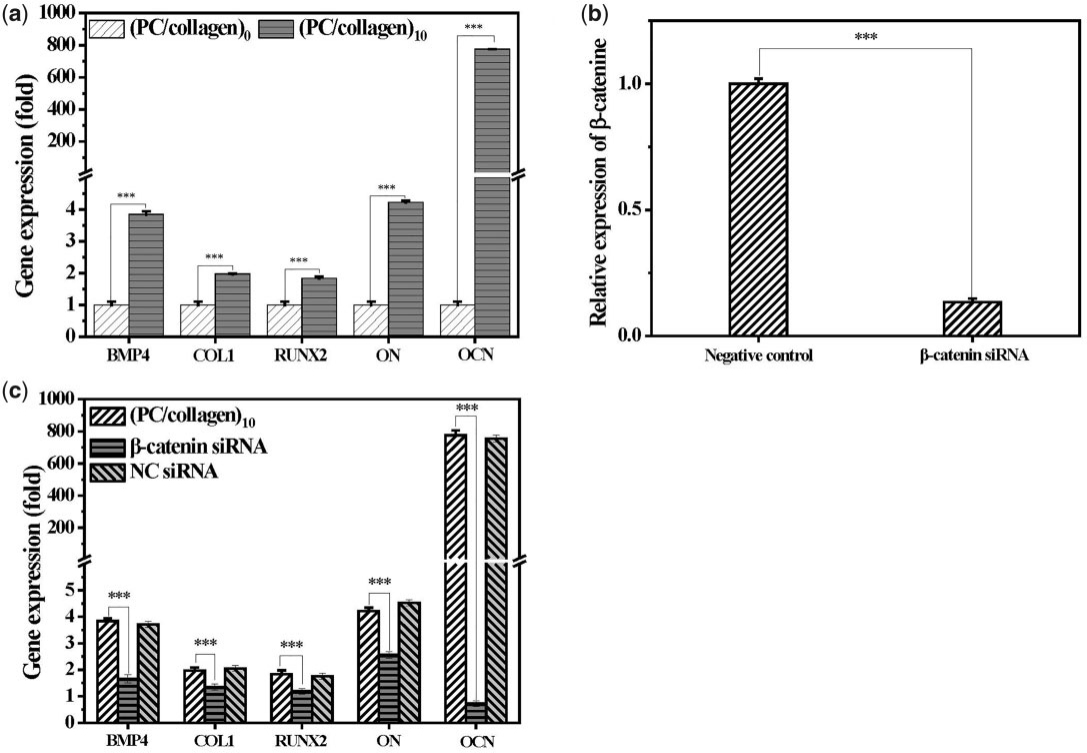
(**a**) The mRNA Level of osteogenesis-related genes in BMSCs cultured in slides without or with coating of (PC/collagen)_10_ for 7 days. (**b**) The mRNA level of β-catenin in BMSCs pretreated by corresponding small interference RNA, with NC siRNA as control. (**c**) Pretreatment of siRNA targeting β-catenin effectively inhibit the upregulation of BMP4, RUNX2, COL1, on and OCN in BMSCs induced by (PC/collagen)_10_ (*n* = 3; *P*-value <0.001 was marked with ***).

To evaluate the effect of this newly designed material on the osteogenic differentiation of BMSCs, the expression of osteogenic-related genes (BMP4, ON, OCN, Col1 and RUNX2) in BMSCs cocultured with or without (PC/collagen)_10_ for 7 days when the slide was almost filled with cells were analyzed by qRT-PCR. It was showed that co-culture with (PC/collagen)_10_ significantly upregulated the mRNA level of BMP4, OCN, ON, RUNX2 and Col1 in BMSCs (Fig. 6a). The newly designed material was revealed to be able to effectively upregulate the genes closely associated with osteogenesis in BMSCs, among which OCN was the most upregulated.

OCN is found highly expressed in bone tissue, specifically in osteoblasts [33] and participates in bone mineralization through carboxylation [34]. Several studies have shown that OCN does not participate in bone formation but affects the longitudinal bone strength by increasing bone energy consumption and plasticity [35, 36]. The internal bone volume of transgenic mice with significantly decreased expression of OCN was also significantly decreased [37]. In the case of a lack of OCN expression, the apatite crystal thickness of the bone in the organism becomes thinner. In this study, the newly synthesized materials upregulated OCN in osteogenesis-related genes, which may promote osteogenesis by regulating bone mineralization and bone strength.

However, the underlying mechanism of promoting osteogenesis of newly designed materials remains unknown. Previous reports have indicated that the Wnt/β-catenin pathways are closely related with the osteogenic differentiation of BMSCs [38]. To explore the potential role of Wnt/β-catenin pathways in the newly designed materials’ upregulation of osteogenic-related gene expression, we used β-catenin siRNA to specifically inhibit the Wnt/β-catenin pathways in BMSCs cocultured with the new materials for 7 days, and then detected the mRNA level of genes associated with osteogenesis. The results showed that silencing β-catenin could effectively inhibit the up-regulation of osteogenesis-related genes in BMSCs by new designed materials (Fig. 6b and c). Therefore, it was reasonable to believe that the newly designed material promotes osteogenesis of BMSCs through activation of Wnt/β-catenin signal pathway. The positive correlation between the activation of Wnt/β-catenin signaling pathway and osteogenic differentiation of stem cells found in this study is consistent with the results previously reported [39–41].

**Figure 7.**
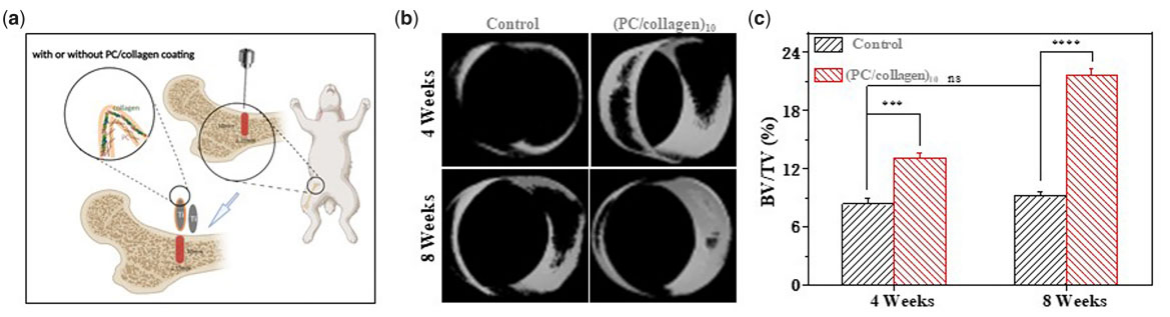
(**a**) Schematic diagram of the experiment *in vivo*. (**b**) Micro-CT was used to detect the qualities of the regenerated bone around the implanted titanium rods containing control or (PC/collagen)_10_ after 4 and 8 weeks. The micro-CT images are shown in (b). (**c**) Comparison of the bone volume fraction (BV/TV) of the implants of (b). (*n* = 3; ns, no significance; *P*-values <0.001 and 0.0001 are marked with *** and ****, respectively) (schematic illustration created with BioRender.com).

Titanium alloy implants are commonly used in orthopedic surgery because of their stiffness and good biocompatibility [42]. However, how to promote the better connection of titanium implants and surrounding bone and promote bone growth on the surface is still a challenge to be resolved [43]. To further verify the osteogenic effect of the materials designed in this study *in vivo*, a titanium rod coated with or without (PC/collagen)_10_ was used to treat five rabbits with bone defects of almost the same area in the femur. When the coated or uncoated titanium rod was implanted in rabbits with bone defects for 4 and 8 weeks, the femur with implant material was collected and tested under micro-CT. Consistent with the results of cytological experiments *in vitro*, the coating material promoted osteogenesis (Fig. 7b and c).

PC/collagen promotes the proliferation of BMSCs through integrin-related signaling pathways and reduces the intracellular ROS. As a surface receptor, integrin can be activated and transmit signals to regulate intracellular gene expression, which in turn induces value addition, survival, migration and differentiation [44]. To mediate the interaction between cells and the ECM, integrin can recruit multiprotein complexes after binding to extracellular ligands and activate signal cascades through intracellular protein kinase and adaptor molecules, including integrin-linked kinase (ILK) transmission, to regulate biochemical processes such as growth, proliferation, differentiation, migration and angiogenesis [45–47]. Collagen type I was significantly upregulated in the BMSCs cultured on the newly designed material. The stimulation of collagen type I can increase the localization of ILK to the plasma membrane and promote the formation of a signaling platform on the cell membrane, which leads to enhanced cell migration and invasion by interacting with the actin cytoskeleton and many signal transduction pathways [48], such as the effective activation of integrin signaling pathways. Integrin can also promote the adhesion of cells to collagen type I, which is also beneficial to the homing and adhesion of BMSCs to bone tissue [49]. A data analysis structure based on cDNA microarray shows that the attachment of collagen type I can provide a proliferative signal for cells and improves energy metabolism to increase the rate of increment [49]. Attachment of collagen type I can also increase the level of calcium in the cytoplasm [48] and promote the expression of some molecules regulated by calcium and cyclic adenosine monophosphate (cAMP) [49]. Under the stimulation of collagen type I, extracellular calcium ions are transferred into cells, resulting in a decrease in extracellular calcium concentration. Calcium-dependent E-cadherin is easily cleaved by proteolysis with a decrease in calcium concentration [50], resulting in the loss of adhesion function and loss of contact between cells [51]. Only then can Mg^++^-dependent α2β1 integrin redistribute to the cell–ECM interface from the lateral membrane surface and activate the integrin signaling pathway, increasing the increment and migration of cells at the collagen type I interface [51]. Upregulated collagen type I can also effectively inhibit the FasL signaling pathway and improve cell death by promoting cell adhesion [52].

Oxidative stress has been reported to be able to inhibit cell proliferation and osteogenic differentiation by upregulating apoptosis-related genes, and reducing ROS has been reported to be beneficial for bone regeneration and osseointegration around the implant [53, 54]. With an excellent antioxidant effect, procyanidins can inhibit the expression of apoptosis-related genes [55], and the function of osteoclasts [56], promoting the survival of osteocytes and the deposition of minerals in bone trabeculae [57].

However, PC/collagen promotes osteogenesis by activating the RNUX2 transcription factor and the Wnt/β-catenin signaling pathway and inhibiting the expression of receptor activator of nuclear factor-κB ligand (RANKL). Collagen can induce osteoblast differentiation by binding to α2β1 integrin and affecting the activity of Runx2, which is the upstream of several osteogenesis related genes [58]. Previous reports have shown that the activation of Wnt/β-catenin signaling pathway is closely related to the osteogenic differentiation of BMSCs, but the potential mechanism remains to be elucidated [59]. The newly designed materials in this study can decrease cell–cell adhesion and promote cell migration, increasing the contact of Wnt with receptors on the cell surface. Excessive accumulation of ROS may lead to oxidation of lipids, which may inhibit the secretion of Wnt and the activation of Wnt/β-catenin signaling pathway [60]. With antioxidant activity, the newly designed material is beneficial for the decreasing of ROS level and activation of Wnt/β-catenin signaling pathway. In the absence of Wnt ligands, β-catenin will be ubiquitinated and degraded by APC complexes in the cytoplasm; in the presence of Wnt ligands, β-catenin is separated from the binding with Axin and GSK3 in the cytoplasm and translocated to the nucleus to start transcription of downstream genes, including those related to osteogenesis differentiation [61, 62].

In addition, collagen can also effectively reduce the expression of RANKL in cells to reduce osteoclast formation. As transmembrane protein, RANKL can directly affect the growth and apoptosis of cells [63]. RANKL is expressed in bone and plays a key role in the differentiation and survival of osteoclasts by interacting with its receptor RANK. With concentration of 50 μg/ml, PC could significantly inhibit RANKL-dependent differentiation of 95% osteoclasts and inhibit mature osteoclasts induced bone degradation by reducing the release of collagen helical peptide [26].

**Figure 8.**
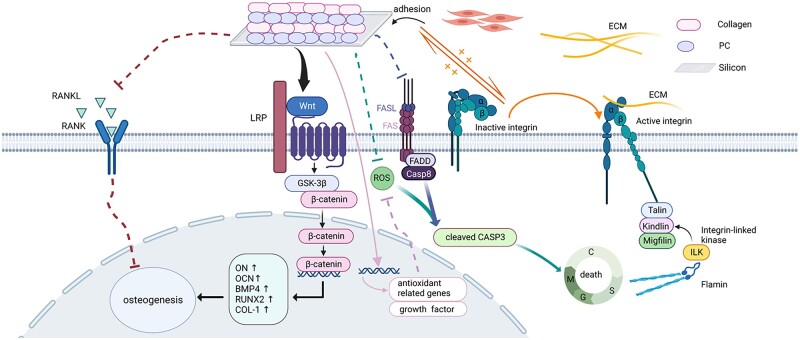
The potential mechanism of the function of newly designed (PC/collagen)_10_ (created with BioRender.com).

In summary, our results show that newly designed (PC/collagen)_10_ coating promotes the proliferation and osteogenic differentiation of BMSCs *in vitro* and is beneficial for new bone formation around the implant *in vivo*, which is probably by reducing the level of ROS, and activation of the Wnt/β-catenin signaling pathway (Fig. 8).

## Conclusions

In short, a series of experiments have proven that PC/collagen multilayers have very important value for research and application as implant coating materials. The PC/collagen multilayer can effectively promote the proliferation of BMSCs and reduce the damage to BMSCs induced by high oxidative stress. These functions enhanced bone generation *in vivo* and the expression of osteogenic-related genes (COL1, BMP4, Runx2, OCN and ON) in BMSCs *in vitro* via activated Wnt/β-catenin pathway. We believe that the discovery of PC/collagen will provide a new direction for the synthesis of coatings for bone-regeneration in the future.

## Supplementary Material

rbac107_Supplementary_Data
