# Supplementary material for: Layer-by-layer assembly of procyanidin and collagen promotes mesenchymal stem cell proliferation and osteogenic differentiation in vitro and in vivo
Source: Regen Biomater. 2022 Dec 26;10:rbac107. doi: 10.1093/rb/rbac107 (PMC9847536; doi:10.1093/rb/rbac107)
Supplement: rbac107_Supplementary_Data [file rbac107_supplementary_data.docx]

**Primer sequences for qRT-PCR**

| Genes | Forward primer sequence | Reverse primer sequence |
| --- | --- | --- |
| COL1 | 5′-GGGGCAAGACAGTCATCGAA-3′ | 5′-GAGGGAACCAGATTGGGGTG-3′ |
| OCN | 5′-AAGCAGGAGGGCAATAAGGT-3′ | 5′-TTTGTAGGCGGTCTTCAAGC-3′ |
| β-catenin | 5′-GGTGGAACGCAGCAGCAGT-3′ | 5′-CCCGAGCAAGGATGTGGAGA-3′ |
| RUNX2 | 5′-AACGATCTGAGATTTGTGGGC-3′ | 5′-CCTGCGTGGGATTTCTTGGTT-3′ |
| ON | 5′-TGAGGACGGTGCAGAGGA-3′ | 5′-TGGTGGCAAAGAAGTGGC-3′ |
| β-actin | 5′- GGCTGTATTCCCCTCCATCG-3′ | 5′-CCAGTTGGTAACAATGCCATGT-3′ |
